# Remote training solutions for complex intervention trials

**DOI:** 10.1186/1745-6215-16-S2-P194

**Published:** 2015-11-16

**Authors:** Rebecca Lewis, Shaista Hafeez, Helen McNair, Joanna Illambas, Charlotte Friend, Emma Hall, Robert Huddart

**Affiliations:** 1 Clinical Trials and Statistics Unit at The Institute of Cancer Research (ICR-CTSU), London, UK; 2 The Institute of Cancer Research, London, UK; 3 The Royal Marsden NHS Foundation Trust, London, UK

## Background

Delivering training to participating sites is an essential part of successful trial setup especially when new techniques, treatments or technologies are involved. RAIDER is a multicentre (> 30 NHS Trusts) phase II trial of complex radiotherapy for muscle invasive bladder cancer. The radiotherapy techniques were developed at a single UK NHS site and in addition to written materials ideally require demonstration using the relevant radiotherapy planning and imaging software, only available at the experienced site. Rather than arranging a series of visits from key participating site personnel (Principal Investigator (PI) and research radiographers), a virtual meeting plan was developed.

## Methods

Cisco’s WebEx was selected as it allowed screen sharing and live demonstration of radiotherapy contouring techniques. Sites were provided with guidelines for using WebEx and given a practice session to check connectivity. The 2 hour interactive meeting included an introduction to the trial, technical details of treatment delivery and opportunities to ask questions. Feedback was requested from attendees.

## Results

132 attendees from 27 sites participated, including 18 PIs. 26/27 sites provided feedback with 20 stating that the virtual meeting allowed more staff to attend compared to a face-to-face meeting; 17/26 preferred the virtual meeting format. The usefulness of the meeting was rated 8.6/10 on average.

## Conclusion

The virtual meeting format improved attendance rates, was considered beneficial by attendees and facilitated recording for future use by staff or sites unable to attend. The approach was a useful training tool for RAIDER and will be adopted by other ICR-CTSU trials.

